# Trajectories of physical activity, from young adulthood to older adulthood, and pancreatic cancer risk; a population-based case-control study in Ontario, Canada

**DOI:** 10.1186/s12885-020-6627-8

**Published:** 2020-02-21

**Authors:** Jaspreet Sandhu, Vanessa De Rubeis, Michelle Cotterchio, Brendan T. Smith, Lauren E. Griffith, Darren R. Brenner, Ayelet Borgida, Steven Gallinger, Sean Cleary, Laura N. Anderson

**Affiliations:** 1 Department of Health Research Methods, Evidence, and Impact, McMaster University, Hamilton, ON Canada; 2 Prevention and Cancer Control, Cancer Care Ontario, Toronto, ON Canada; 3 Dalla Lana School of Public Health, University of Toronto, Toronto, ON Canada; 4 Public Health Ontario, Toronto, ON Canada; 5 Alberta Health Services, Cancer Control, Calgary, AB Canada; 6 Department of Oncology and Community Health Sciences, Cumming School of Medicine, University of Calgary, Calgary, AB Canada; 7 Lunenfeld-Tanenbaum Research Institute, Mount Sinai Hospital, Toronto, ON Canada; 8 Division of General Surgery, Toronto General Hospital, Toronto, ON Canada; 9 Department of Surgery, University Health Network, University of Toronto, Toronto, ON Canada; 10 Division of Hepatobiliary and Pancreas Surgery, Mayo Clinic, Rochester, MN USA

**Keywords:** Physical activity, Life-course, Trajectory, Pancreatic cancer

## Abstract

**Background:**

There is inconsistent evidence on the association between physical activity and pancreatic cancer risk and few studies have investigated early life or life-course physical activity. The objective of this study was to evaluate the association between trajectories of physical activity across the life-course and pancreatic cancer risk.

**Methods:**

A population-based case-control study was conducted (2011–2013) using cases (*n* = 315) from the Ontario Pancreas Cancer Study and controls (*n* = 1254) from the Ontario Cancer Risk Factor Study. Self-reported recall of moderate and vigorous physical activity was measured at three time points: young adulthood (20s–30s), mid-adulthood (40s–50s) and older-adulthood (1 year prior to questionnaire completion). Physical activity trajectories were identified using latent class analysis. Odds ratios (OR) and 95% confidence intervals (CI) were estimated from multivariable logistic regression adjusted for covariates: age, sex, race, alcohol, smoking, vegetable, fruit and meat consumption, and family history of pancreatic cancer.

**Results:**

Six life-course physical activity trajectories were identified: inactive at all ages (41.2%), low activity at all ages (31.9%), increasingly active (3.6%), high activity in young adulthood with substantial decrease (13.0%), high activity in young adulthood with slight decrease (5.0%), and persistent high activity (5.3%). Compared to the inactive at all ages trajectory, the associations between each trajectory and pancreatic cancer after confounder adjustment were: low activity at all ages (OR: 1.11; 95% CI: 0.75, 1.66), increasingly active (OR: 1.11; 95% CI: 0.56, 2.21), high activity in young adulthood with substantial decrease in older adulthood (OR: 0.76; 95% CI: 0.47, 1.23), high activity in young adulthood with slight decrease in older adulthood (OR: 0.98; 95% CI: 0.62, 1.53), and persistently high activity (OR: 1.50; 95% CI: 0.86, 2.62). When time periods were evaluated separately, the OR for the association between high moderate activity in the 20s–30s and pancreatic cancer was 0.89 (95% CI: 0.64, 1.25) and some sex differences were observed.

**Conclusion:**

Distinct life-course physical activity trajectories were identified, but there was no evidence that any of the trajectories were associated with pancreatic cancer. Future studies with larger sample sizes are needed to understand the associations between physical activity trajectories over the life-course and pancreatic cancer risk.

## Background

Pancreatic cancer remains one of the most deadly forms of cancer, with a very poor prognosis, evidenced by a similar rate between disease incidence and mortality [1]. According to the Canadian Cancer Society, an estimated 5500 Canadians were diagnosed with pancreatic cancer and 4800 died from the disease in 2017 [2]. The case-to-fatality ratio for pancreatic cancer is reported to be 93%, highest among solid tumors in Canada [3]. In Canada, the age-standardized 5-year relative survival was estimated to be approximately 9% [3]. The poor prognosis is largely attributed to the late stage at which most patients are diagnosed, as the disease often remains asymptomatic until advanced stages [1]. The total deaths from pancreatic cancer are rising in both North America and globally, with pancreatic cancer expected to become the second leading cause of cancer death in the USA by 2030 [1].

The incidence of pancreatic cancer varies across different regions and populations suggesting a multi-factorial aetiology of the disease including genetics, lifestyle, and environmental factors [4]. Physical activity is a modifiable lifestyle factor that has been shown to decrease the risk of various types of cancer, with the strongest evidence for decreased risk associated with cancers of the colon, breast, and endometrium [5]. However, there is limited evidence supporting an association between higher physical activity and decreased pancreatic cancer [6–10]. Two systematic reviews showed a possible inverse protective association between total physical activity and occupational physical activity with pancreatic cancer [6, 7], while others have shown this association with leisure-time physical activity [8, 9].

The timing of physical activity over the life-course has been the subject of studies to better understand physical activity in mitigating risk of other diseases, including some cancers [6]. Various models have been proposed in the field of life-course epidemiology including the sensitive-periods model, which suggests that there is a time period when an exposure has a stronger impact on disease risk than it would at other times, and the accumulation of risk model, which suggests that cumulative exposures during the life-course impact the risk of health later in life, regardless of their timing [11]. A systematic review found a small but statistically significant association between leisure-time physical activity and risk of pancreatic cancer (pooled RR: 0.89; 95% CI: 0.83, 0.96) [8]. Another study provides some limited support for an accumulation of risk model showing weak evidence for reduced pancreatic cancer risk with consistent physical activity over time [7]. A recent systematic review identified unique trajectories of physical activity over the life-course [12]. To the best of our knowledge, no study has explicitly examined whether the duration, timing and trajectories of physical activity across a person’s life course are associated with incidence of pancreatic cancer, or explicitly evaluated the impacts of earlier life physical activity on the risk of development of pancreatic cancer. An increasingly utilized approach to understand life-course exposures is the use of trajectory modelling [13–15]. Few studies [16–18] have used this approach to understand the impact of physical activity across the life-course and disease outcomes in adulthood.

The primary objective of the current study was to evaluate the association between trajectories of life-course physical activity and pancreatic cancer risk. As a secondary objective, this study aims to investigate whether earlier adult life is a sensitive period in which higher physical activity mitigates the risk of development of pancreatic cancer.

## Methods

### Study design

A population-based case-control study was conducted using cases from the Ontario Pancreas Cancer Study (OPCS) and controls from the Ontario Cancer Risk Factor Study (OCRF). A detailed description of the study design and data collection are available elsewhere [15, 19]. Briefly, pancreatic cancer cases were recruited by the OPCS between 2011 and 2013. The Ontario Cancer Registry was used to identify pancreatic cancer cases. This population-based registry uses rapid-case ascertainment through electronic pathology reports to collect data from regional cancer centres, hospital discharges and ambulatory care records, and Ontario death certificates for all cancer cases across Ontario. Ontario residents with a pathologically confirmed adenocarcinoma of the pancreas or adenocarcinoma metastasis diagnosed by a physician (International Classification of Diseases for Oncology Third Edition codes C25.0–25.9, with 25.4 neuroendocrine pancreas excluded) were eligible for inclusion into the study. Population-based controls were recruited by the OCRF in 2011 through modified random digit dialing of Ontario households. The population-based controls were frequency matched (3:1) on 5-year age and sex groups based on the expected distribution of cases.

### Sample size and response rates

**Fig. 1:**
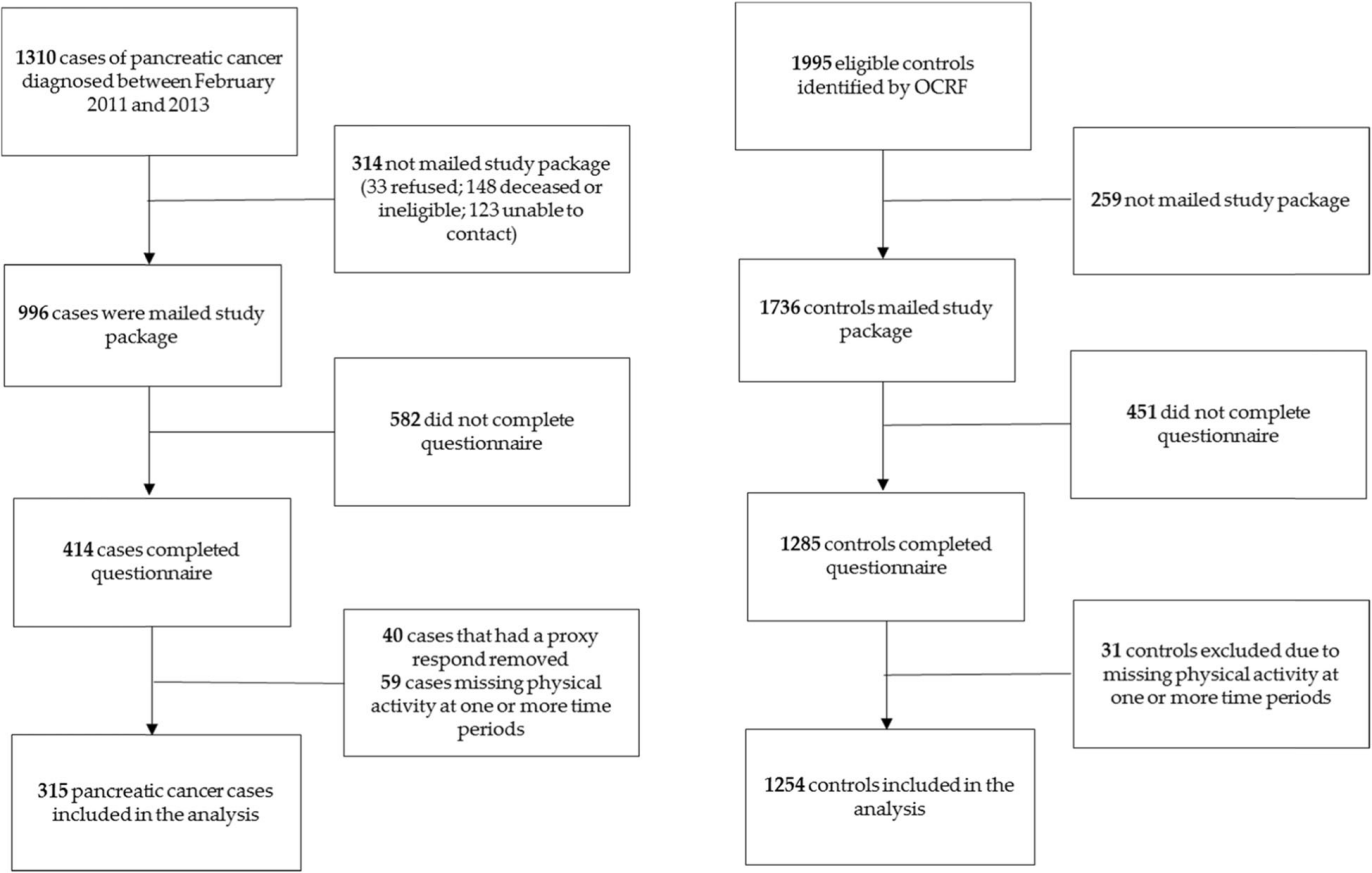
Sampling flow diagram for cases from the Ontario Pancreas Cancer Study (OPCS), and controls from the Ontario Cancer Risk Factor (OCRF) Study

A total of 1310 cases of pancreatic cancer were diagnosed between February 2011 and January 2013, and of these, 314 (24%) were not mailed the study package (33 refused, 158 deceased or ineligible, and 123 unable to contact). Of the 996 that were mailed the questionnaire packages, completed questionnaires were received from 414 (42%) participants. However, 40 cases with proxy respondents and 59 cases missing physical activity at one or more time periods were excluded from the analysis. A total of 315 pancreatic cases were included in the analysis. A total of 1995 eligible controls were identified by the OCRF. The study package was mailed to 1736 (87%) who agreed to participate. The epidemiologic questionnaire was completed by 1285 (74%) participants, however 31 controls were excluded due to missing physical activity data at one or more time points, leaving 1254 controls included within the analysis of this study. Figure 1 displays the sampling flow chart.

### Research ethics

Research ethics approval was obtained from the University of Toronto and Mount Sinai Hospital, Toronto, Canada, for the primary data collection. For the current study, which included secondary data analysis of de-identified data, research ethics approval was received from Hamilton Integrated Research Ethics Board (HiREB), Hamilton, Canada.

### Measurement of physical activity

Participants were mailed a study package which included self-administered questionnaires that asked them to report their physical activity with the question “During your 20s and 30s, how often did you take part in moderate physical activity (such as bowling, golf, light sports, physical exercise, gardening, taking long walks, or while at work)?”. A similar question was asked to identify vigorous physical activity, “During your 20s and 30s, how often did you take part in vigorous physical activity (such as jogging, racquet sports, swimming, aerobics, strenuous sports, or while at work)?”. Physical activity was reported for three timepoints; young adulthood (20s and 30s), mid-adulthood (40s and 50s) and 2 years ago (i.e., 2 years prior to completion of the questionnaire). When reporting physical activity participants were given four options: rarely/never, a few times per month (1/week), 2–4 times per week, or > 4 times per week. Participants were advised to include both leisure and work activity together during each time period.

Moderate and vigorous physical activity are reported separately for each timepoint (20s and 30s, 40s and 50s, and 2 years ago). All participants had the option to respond to each timepoint, although for some participants 2 years ago would also be in 40s and 50s. A total cumulative physical activity score (METs/week) was derived for each time period, combining moderate and vigorous activity. The number of times of physical activity per week was multiplied by an average metabolic equivalent of task (MET) score. An average MET score of 7 was used for vigorous activity, and a score of 3 was used for moderate activity. These average MET scores were chosen based on the characterization of moderate and vigorous intensity in the literature [20]. An overall total physical activity score was created by taking the sum of physical activity across all timepoints measured in MET score/week.

### Measurement of other variables

Assessment of all other variables was collected via self-reported mailed questionnaires 2 years prior to cancer diagnoses for cases or 2 years earlier for controls. Variables were selected a priori for inclusion in the models if they were considered to be potential confounders (i.e., associated with both the exposure, physical activity, and the outcome, pancreatic cancer, but not on the causal path [21]). Age, sex, education, race, alcohol intake, smoking, fruit, vegetable and meat consumption, and family history of pancreatic cancer were included in the fully adjusted model as potential confounding variables [22, 23]. Diabetes, pancreatitis and current body mass index (BMI) were not included in the adjusted model as they were hypothesized to potentially be on the causal path between physical activity and pancreatic cancer. A third analyses was run that included these three variables in additional to the potential confounding variables Education was categorized as high school graduate or less, and college/university graduate. Alcohol consumption was categorized as never, former, current light to moderate drinker (1–20 drinks/week) and current heavy drinker (> 21 drinks/week). Smoking was included in the model as a categorized pack-years variable. This variable was derived from the number of years an individual smoked and the average number of cigarettes smoked per day.

### Defining physical activity trajectories

A group-based trajectory modelling approach was used to define the physical activity trajectories in the statistical software, SAS 9.4 [24]. PROC TRAJ, is a statistical package that is available free of charge for download (www.andrew.cmu.edu/user/bjones/) to implement in SAS for group-based trajectory modeling [25]. Using this group-based trajectory modelling procedure we identified distinct subgroups (or clusters) among the study population which shared underlying trajectories of physical activity. This method allowed us to identify discrete trajectories of physical activity longitudinally over the life-course [26]. Data from all three time points of physical activity (20s and 30s, 40s and 50s, and 2 years prior) were used to define the trajectories using the cumulative measure that combined moderate and vigorous activity (METs/week).

Trajectories were generated by consulting literature by Nagin [26] and following the proposed framework by Lennon et al. [27]. We first identified the potential number of trajectories that may fit the model based on previous literature. A recent systematic review noted the most common number of trajectories of physical activity across the life-course were 3–5 [12]. We tested models with up to 7 trajectories. The optimal model fit was determined based on the lowest Bayesian Information Criterion (BIC) across the various models. Significance of polynomial terms were also used to assess goodness-of-fit. Next, we calculated the average posterior probability, using a cut-off value of 0.70 [25].

It is recommended, all trajectories hold a minimum of 5% group membership [28], however the increasingly active group held 3.6% of the study sample. When decreasing the number of classes within the model, this group remained so we retained all six trajectories. A six-class trajectory was determined to be the best model to fit this data. In accordance with studies of similar methodologies [29] and upon visual inspection, each trajectory was given a name.

### Statistical analysis

All statistical analyses were conducted using the statistical software SAS 9.4 [24] with the PROC TRAJ package. Descriptive statistics were calculated for all variables for both cases and controls. We used unconditional multivariable logistic regression to estimate adjusted odds ratios (OR) with 95% confidence intervals (CI) for physical activity at separate time-points and physical activity trajectories across the life-course and pancreatic cancer risk. Results for two models are presented: 1) a parsimonious model adjusted only for age and sex; 2) a fully adjusted model that included age, sex, and all potential confounders. Age and sex were adjusted for in all models to account for frequency matching. We conducted sensitivity analysis where we included the potential mediating variables (diabetes, BMI and pancreatitis) in the fully adjusted model, however, results were similar to the fully adjusted model and are not shown here. All analyses were stratified by sex to determine any differences.

## Results

### Descriptive characteristics

Characteristics of the study participants and known pancreatic cancer risk factors are described in Table 1 and have been described previously [19]**.** Controls were matched to cases on sex and expected age group distribution and 49% of cases and 47% of controls were female. 40% of cases and 46% of controls had a university or college degree and 14% of cases and 8% of controls were non-Caucasian. Established pancreatic risk factors including family history of pancreatic cancer (OR: 3.16; 95% CI:1.97, 5.06) and ever smoking (OR: 1.29; 95% CI:1.00, 1.67) were associated with increased odds of pancreatic cancer (Table 1).

**Table 1:** Age group and sex-adjusted odds ratio estimates for pancreas cancer risk factors among Cases and Controls from Ontario, Canada (*n* = 1569)

Characteristic	Cases (*N* = 315) N	%	Controls (*N* = 1254) N	%	OR^a^ (95% CI)
Family History of Pancreas Cancer^b^					
No	261	83	1144	91	1.00
Yes	33	11	46	4	3.16 (1.97, 5.06)
Don’t know	21	7	60	5	
Missing	0	0	4	0.3	
Cigarette Smoking					
Never	123	39	569	45	1.00
Ever	190	60	684	55	1.29 (1.00, 1.67)
Missing	2	1	1	0.1	
Alcohol Consumption^c^					
Never	107	34	403	32	1.00
Former	26	8	88	7	1.14 (0.69, 1.86)
Current	181	57	754	60	0.92 (0.69, 1.21)
Missing	0	0.7	9	1	
Body Mass Index (kg/m^2^)^d^					
< 25.0	103	33	399	32	1.00
25.0- < 30.0	107	34	512	41	0.84 (0.62, 1.14)
≥ 30.0	101	32	336	27	1.24 (0.90, 1.71)
Missing	4	1	7	1	
Ethnicity					
Caucasian	268	85	1153	92	1.00
Other	45	14	98	8	1.97 (1.35, 2.88)
Missing	2	0.6	3	0.2	
Education^e^					
College/University graduate	127	40	571	46	1.00
High school graduate or less	185	59	679	54	1.17 (0.91, 1.51)
Missing	3	1	4	0.3	
Gender					
Male	162	51	666	53	–
Female	153	49	588	47	–
Age (y)^f^					
< 60	81	26	439	35	–
60–64	64	20	285	23	–
65–69	63	20	218	17	–
≥ 70	104	33	312	25	–
Missing	3	1	0	0	–

a. Age group and sex adjusted OR

b. First degree relatives

c. Approximately 2 years prior to questionnaire completion

d. One year before questionnaire completion

e. Highest level of education reached

f. Age at pancreas cancer diagnosis for cases; age at questionnaire completion for control

### Trajectories of physical activity over the life-course

**Fig. 2:**
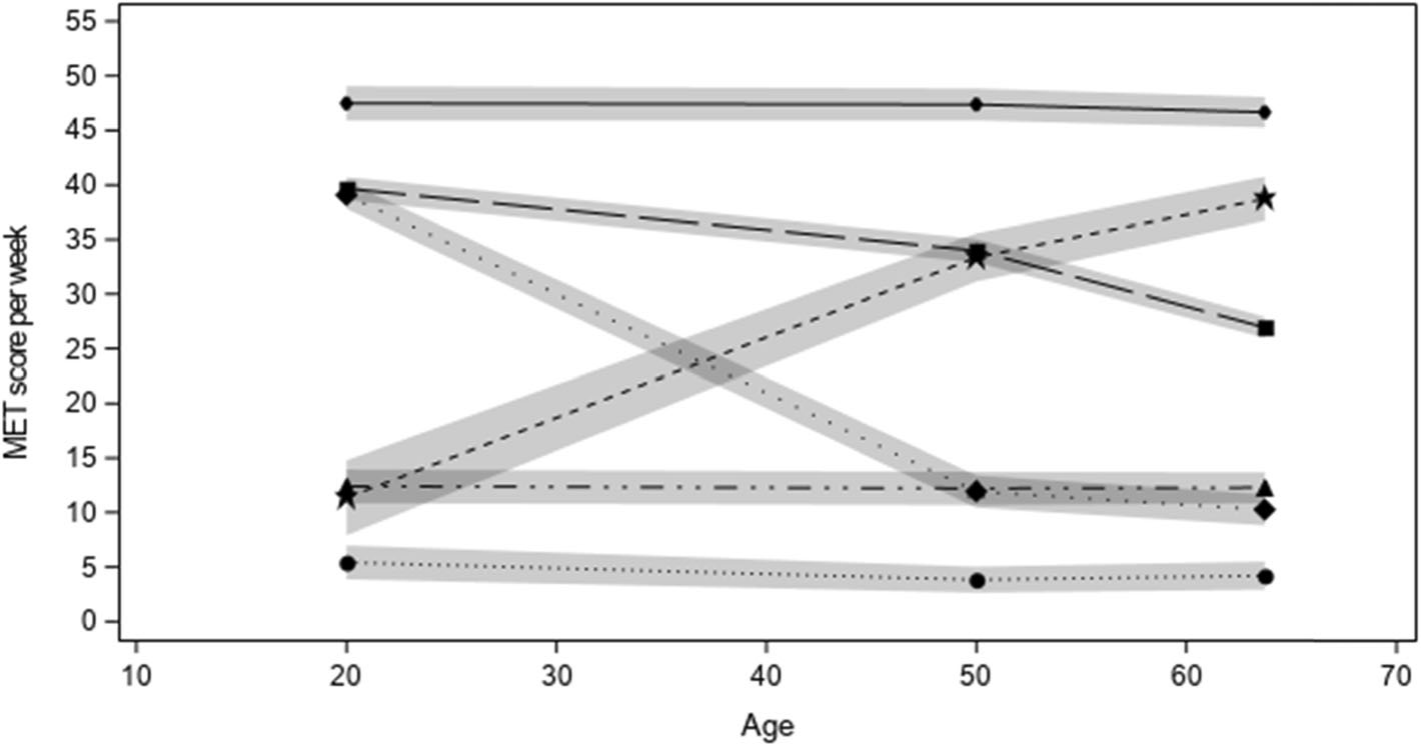
Trajectories of physical activity over the life-course (*n* = 1569) among Cases and Controls from Ontario, Canada

The trajectory modeling identified six distinct physical activity trajectories across the life-course (Fig. 2): inactive at all ages (16.7%), low activity at all ages (33.7%), increasingly active (4.8%), high activity in young adulthood with substantial decrease (16.4%), high activity in young adulthood with slight decrease (20.1%), and persistent high activity (8.1%). These trajectories were labelled based on visual assessment of the model.

The OR and 95% CI for the association between each identified trajectory and odds of pancreatic cancer are provided in Table 2. Compared to the inactive at all ages trajectory (reference group), the ORs with pancreatic cancer for each trajectory were: low activity at all ages, adjusted OR: 1.11(95% CI: 0.75, 1.66), increasingly active, adjusted OR: 1.11 (95% CI: 0.56, 2.21), high activity in young adulthood with slight decrease in older adulthood, adjusted OR: 0.98 (95% CI: 0.62, 1.53), high activity in young adulthood with substantial decrease in older adulthood, adjusted OR: 0.76 (95% CI: 0.47, 1.23), and persistent high activity, adjusted OR: 1.50 (95% CI: 0.86, 2.62). None of the ORs changed substantially when BMI, diabetes and pancreatitis were included, in addition to the other variables, in the fully adjusted model (results not shown). When stratified by sex, possible differences between males and females were observed across various physical activity trajectories and pancreatic cancer risk (Table 3). For example, the adjusted OR for the association between the ‘high activity in young adulthood with slight decrease in older adulthood’ trajectory and pancreatic cancer among males was1.35 (95% CI: 0.72, 2.51) and for females the adjusted OR was 0.57 (95% CI: 0.27, 1.21). Similarly, for the “increasingly active” trajectory in males the adjusted OR was 2.53 (95% CI: 0.89, 7.20), whereas in females the adjusted OR was 0.62 (95% CI: 0.24, 1.61). However, none of these sex stratified associations were statistically significant at *p* < 0.05 and confidence intervals were very wide and overlapped 1.0.

**Table 2:** Odds ratio estimates for physical activity trajectories across life-course and pancreatic cancer risk among Cases and Controls from Ontario, Canada

Age-specific physical activity trajectories	Cases *N* = 315	%	Controls *N* = 1254	%	OR^a^ (95% CI)	OR^b^ (95% CI)
Group 1: Inactive at all ages	56	18	196	16	1.00	1.00
Group 2: Low activity at all ages	107	34	411	33	0.94 (65, 1.35)	1.11 (0.75, 1.66)
Group 3: High activity in young adulthood with slight decrease in older adulthood	61	19	264	21	0.80 (0.53, 1.21)	0.98 (0.62, 1.53)
Group 4: Increasingly active	15	5	57	5	0.95 (0.50, 1.80)	1.11 (0.56, 2.21)
Group 5: High activity in young adulthood with substantial decrease in older adulthood	44	14	237	19	0.71 (0.45, 1.10)	0.76 (0.47, 1.23)
Group 6: Persistent high activity	32	10	89	7	1.28 (0.77, 2.14)	1.50 (0.86, 2.62)

a. Age group and sex adjusted OR

b. Age group, sex, alcohol consumption, smoking, vegetable consumption, fruit consumption, red meat consumption, family history of pancreatic cancer, race, education adjusted OR

**Table 3:** Odds ratio estimates for physical activity trajectories across life-course and pancreatic cancer risk among Cases and Controls from Ontario, Canada stratified by sex

Age-specific physical activity trajectories	Males				Females			
	Cases *N* = 162%	Controls *N* = 666%	OR^a^ (95% CI)	OR^b^ (95% CI)	Cases *N* = 153%	Controls *N* = 588%	OR^a^ (95% CI)	OR^b^ (95% CI)
Group 1: Inactive at all ages	13	14	1.00	1.00	23	18	1.00	1.00
Group 2: Low activity at all ages	28	28	1.10 (0.62, 1.95)	1.38 (0.74, 2.57)	40	38	0.83 (0.51, 1.33)	0.94 (0.55, 1.64)
Group 3: High activity in young adulthood with slight decrease in older adulthood	27	27	1.10 (0.61, 1.96)	1.35 (0.72. 2.51)	12	15	0.53 (0.27, 1.03)	0.57 (0.27, 1.21)
Group 4: Increasingly active	5	3	1.99 (0.76, 5.20)	2.53 (0.89, 7.20)	5	7	0.54 (0.22, 1.34)	0.62 (0.24, 1.61)
Group 5: High activity in young adulthood with substantial decrease in older adulthood	14	20	0.78 (0.41, 1.51)	0.90 (0.45, 1.81)	14	18	0.68 (0.37, 1.25)	0.71 (0.35, 1.41)
Group 6: Persistent high activity	13	9	1.57 (0.78, 3.17)	1.78 (0.83, 3.80)	7	5	1.01 (0.46, 2.25)	1.24 (0.51, 3.04)

a. Age group adjusted OR

b. Age group, alcohol consumption, smoking, vegetable consumption, fruit consumption, red meat consumption, family history of pancreatic cancer, race, education adjusted OR

### Physical activity and pancreatic cancer at different periods of life

The associations between moderate and vigorous physical activity and pancreatic cancer separately for each time period over the life-course are provided in Tables 4 and 5, respectively. Results are provided for the total study population and stratified by sex. None of the associations between moderate physical activity and pancreatic cancer were statistically significant at any age period (Table 4), but there was some possible evidence of sex differences. Similarly, for vigorous physical activity at each of the time periods, nearly all associations, overall and stratified by sex, were not statistically significant (Table 5). Among the total study population, those who exercised a few times per month had reduced odds of pancreatic cancer in comparison to those who rarely/never exercised (OR: 0.64; 95% CI: 0.44, 0.92), but there was no consistent dose-response relationship with increasing activity levels. Among females the adjusted ORs were consistently less than 1.0 for all frequencies of exposure and at each age period, whereas for males many of the OR were closer to 1.0 and in the case of the highest frequency of activity (> 4 times per week) the OR were consistently greater than 1.0. For example, among males vigorous intensity physical activity > 4 times per week during 40s and 50s (OR: 1.62; 95% CI: 0.95, 2.76) and 2 years prior to completion of questionnaire (OR: 1.67; 95% CI: 0.94, 2.95) were possibly associated with increased odds of pancreatic cancer (Table 5). The associations between moderate and vigorous physical activity at individual timepoints and pancreatic cancer risk were further stratified by age of study participants (greater than or less than 65 years) and the stratified results did not reveal any obvious effect modification (see supplemental Tables S1 and S2). None of the interactions between either sex or age group and any of the physical activity measures were statistically significant at *p* < 0.05.

**Table 4:** Odds ratio estimates for moderate physical activity levels throughout the life-course among Cases and Controls from Ontario, Canada stratified by sex ^a^

Physical activity levels for various periods	Total population	MALE			FEMALE		
	Adjusted OR^b^ (95% CI)	Cases *N* = 162 (%)	Controls *N* = 666 (%)	Adjusted OR^c^ (95% CI)	Cases *N* = 153 (%)	Controls *N* = 588 (%)	Adjusted OR^c^ (95% CI)
Moderate activity level at age 20s and 30s							
Rarely/Never or a few times per month	1.00	20	23	1.00	10	27	1.00
2–4 times per week	0.98 (0.69, 1.39)	36	31	1.43 (0.86, 2.41)	27	35	0.65 (0.39, 1.08)
> 4 times per week	0.89 (0.64, 1.25)	44	46	1.03 (0.63, 1.69)	37	38	0.75 (0.46, 1.22)
Moderate activity level at ages 40s and 50s							
Rarely/Never or a few times per month	1.00	35	30	1.00	35	29	1.00
2–4 times per week	1.12 (0.80, 1.56)	36	37	1.46 (0.90, 2.37)	34	38	0.87 (0.55, 1.41)
> 4 times per week	1.12 (0.79, 1.59)	38	33	1.39 (0.98, 2.59)	29	31	0.71 (0.42, 1.20)
Age not reached		2	1		2	2	
Moderate activity level 2 years ago							
Rarely/Never or a few times per month	1.00	20	23	1.00	25	24	1.00
2–4 times per week	1.09 (0.75, 1.56)	33	34	1.24 (0.74, 2.10)	39	41	0.94 (0.56, 1.59)
> 4 times per week	1.33 (0.93, 1.90)	48	43	1.51 (0.92, 2.47)	37	35	1.17 (0.69, 1.99)

a. All interaction terms with physical activity, age and sex were not statistically significant

b. Age group, sex, alcohol consumption, smoking, vegetable consumption, fruit consumption, red meat consumption, family history of pancreatic cancer, race, education adjusted OR

c. Age group, alcohol consumption, smoking, vegetable consumption, fruit consumption, red meat consumption, family history of pancreatic cancer, race, education adjusted OR

**Table 5:** Odds ratio estimates for vigorous physical activity levels throughout the life-course among Cases and Controls from Ontario, Canada stratified by sex ^a^

Physical activity levels for various periods	Total population	MALES			FEMALES		
	Adjusted OR^b^ (95% CI)	Cases *N* = 162 (%)	Controls *N* = 666 (%)	Adjusted OR^c^ (95% CI)	Cases *N* = 153 (%)	Controls *N* = 588 (%)	Adjusted OR^c^ (95% CI)
Vigorous activity level at age 20s and 30s							
Rarely/Never	1.00	17	15	1.00	40	31	1.00
A few times per month	0.82 (0.57, 1.19)	27	28	0.94 (0.53, 1.69)	26	30	0.70 (0.43, 1.15)
2–4 times per week	0.79 (0.54, 1.17)	25	29	0.93 (0.52, 1.67)	18	22	0.68 (0.38, 1.20)
> 4 times per week	0.88 (0.60, 1.30)	32	28	0.97 (0.55, 1.72)	16	17	0.71 (0.39, 1.29)
Vigorous activity level at ages 40s and 50s							
Rarely/Never	1.00	31	30	1.00	48	44	1.00
A few times per month	0.87 (0.61, 1.23)	17	28	0.83 (0.50, 1.38)	25	25	0.99 (0.60, 1.62)
2–4 times per week	0.83 (0.57, 1.20)	22	28	0.81 (0.49, 1.35)	17	19	0.87 (0.50, 1.52)
> 4 times per week	1.23 (0.81, 1.87)	22	13	1.62 (0.95, 2.76)	9	10	0.69 (0.33, 1.45)
Age not reached		2	0.9		2	2	
Vigorous activity level 2 years ago							
Rarely/Never	1.00	46	41	1.00	64	53	1.00
A few times per month	0.64 (0.44, 0.92)	20	30	0.71 (0.43, 1.16)	14	23	0.55 (0.30, 1.00)
2–4 times per week	0.91 (0.61, 1.34)	17	19	0.96 (0.57, 1.64)	14	15	0.84 (0.46, 1.53)
> 4 times per week	1.33 (0.85, 2.06)	40	10	1.67 (0.94, 2.95)	8	9	0.89 (0.42, 1.87)

a. All interaction terms between physical activity, age, and sex were not statistically significant

b. Age group, sex, alcohol consumption, smoking, vegetable consumption, fruit consumption, red meat consumption, family history of pancreatic cancer, race, education adjusted OR

c. Age group, alcohol consumption, smoking, vegetable consumption, fruit consumption, red meat consumption, family history of pancreatic cancer, race, education adjusted OR

### Cumulative physical activity

The results from a derived cumulative life-course physical activity score are provided in Table 6. The continuous score per one unit increase in METs/week was not associated with odds of pancreatic cancer (adjusted OR: 1.00; 95% CI: 0.99, 1.01). When the score was divided into quartiles, it showed no significant association between total cumulative life-course physical activity and risk of development of pancreatic cancer. For example, the adjusted odds ratio for the highest quartile of the cumulative physical activity score compared to the lowest quartile was OR: 1.14 (95% CI: 0.77, 1.67).

**Table 6:** Cumulative life course physical activity score and risk of pancreatic cancer among Cases and Controls from Ontario, Canada

Cumulative life-course physical activity score^a^	OR^b^ (95% CI)	OR^c^ (95% CI)
Continuous variable	1.00 (1.00, 1.00)	1.00 (0.99, 1.01)
Quartiles		
1 (lowest)	1.00	1.00
2	0.96 (0.67, 1.35)	1.13 (0.78, 1.64)
3	0.82 (0.57, 1.17)	0.90 (0.91, 1.31)
4	1.01 (0.71, 1.44)	1.14 (0.77, 1.67)

a. Total cumulative physical activity was derived by multiplying frequency of physical activity per week by the average MET score for the intensity of physical activity; the sum of the intensities at each timepoint was then taken

b. Age group and sex adjusted OR

c. Age group, sex, alcohol consumption, smoking, vegetable consumption, fruit consumption, red meat consumption, family history of pancreatic cancer, race, education adjusted OR

## Discussion

To the best of our knowledge, the results of this study are the first to describe life-course physical activity trajectories and the association with pancreatic cancer risk. Limited research has indicated a possible association between physical activity during the early life time period only or non-trajectory based measures of cumulative physical activity on pancreatic cancer risk [6, 7], which is somewhat consistent with our results for moderate physical activity, but not vigorous. Overall, our study results are largely inconclusive as the 95% CI for all reported OR were very wide due to low statistical power, but the magnitude and direction of the ORs may warrant further investigation with a larger sample size. For example, the ORs for the two of the life-course trajectories characterized by high physical activity in early life were less than 1.0 possibly suggesting protective effects compared to other trajectories. However, contrary to our hypothesis, the persistent high physical activity trajectory was not associated with a decreased risk of pancreatic cancer and the ORs were suggestive of possible increased risk, particularly among males. The cumulative physical activity across the life-course was not significantly associated with the odds of pancreatic cancer and all OR were close to null.

A recent systematic review [12] found most studies identified three to five physical activity trajectories, which differs from the 6 distinct life-course trajectories identified in the current study. The six identified trajectories reflect plausible experiences of physical activity level throughout the life-course. Understanding life-course trajectories is an important epidemiological consideration, as it may provide insight into sensitive periods of life in which an exposure may have the most significant impact on the development of a disease [11, 30]. These sensitive periods would not be perceptible when only considering cumulative impacts. While our study did not find any such association, it provides methodologies that may be important future life-course epidemiological studies.

Although two previously conducted systematic review and meta-analyses [8, 9] identified statistically significant risk reductions with physical activity and pancreatic cancer, two additional meta-analyses [6, 7] had results which were consistent with our current study, as these studies did not find a significant association between total physical activity and pancreatic cancer. Behrens et al., found consistent physical activity over a period of time to potentially contribute to risk reduction of pancreatic cancer (RR: 0.86; 95% CI: 0.76, 0.97) [7], however, these results are not similar to the findings of our study, as Group 6: Persistent high activity trajectory had an inverse association with pancreatic cancer risk. Overall, results across the published systematic reviews and meta-analyses have very inconsistent results which may be explained to some degree by different measures of physical activity. A recent study reported possible differences by sex when studying physical activity in adolescence and adulthood and risk of pancreatic cancer [31]. These results are consistent with our current study that suggested possible sex differences. Future studies may want to further research how sex modifies the association between physical activity throughout the life-course and pancreatic cancer.

It is a limitation of our study that physical activity was collected based on self-reported recall instead of objective measures such as accelerometry. The lack of objective measurement may introduce measurement error due to the simplified nature of the self-reported assessment via questionnaire. The use of an objective measure such as an accelerometers, pedometers or heart-rate monitors may enhance the accuracy and precision of measurement [32]. However, other studies that have used similar self-reported measures to assess physical activity, have provided some possible evidence that increased physical activity may be associated with a reduction of risk of pancreatic cancer [33–35]. Nonetheless, in such epidemiological studies, using self-reported recall may be the only feasible option. Although self-reported recall of physical activity has been found to be a relatively valid measure [36–40], recalling physical activity at earlier periods of life may introduce additional measurement error. Future studies would benefit from prospective assessments of physical activity, which may decrease the risk of bias associated with recall. Further, we cannot rule out the possibility of recall bias leading to differential measurement error which may result in either over- or under-estimation of the true association. Survival bias may also be a concern, since the disease of interest is one with high fatality although every effort was made to recruit cases shortly after diagnosis through the Ontario Cancer Registry’s rapid-case ascertainment system. Similarly, low response rate and possibility of sampling bias may also threaten study validity. Future studies would benefit from a larger sample size with more statistical power.

Strengths of this study include the population-based sampling strategy used to recruit cases and controls. The detailed nature of the questionnaire allowed for a comprehensive assessment of physical activity across the life-course in terms of frequency and intensity, and a wide range of potential confounders. The controls in this study have previously been compared to data from the Canadian Community Health Survey (CCHS) [15] and were found to be somewhat representative of the general population in Ontario, Canada. We comprehensively assessed a range of potential confounders and known pancreatic cancer risk factors, yet there still may be residual confounding due to measurement error or other unmeasured confounders. Due to privacy issues, data on participant occupation was not made available, and therefore not controlled for in our study. It is possible that certain occupations, in which individuals are exposed to carcinogenic substances may also be physically demanding and this may have contributed to the observed inverse association between trajectories characterized by higher levels of physical activity and pancreatic cancer risk. We also did not have available data on early life physical activity (prior to age 20) which may limit the findings of this study. Without these data, evaluating a sensitive period of growth and development that impact risk of pancreatic cancer may be limited.

## Conclusion

Understanding the cumulative effect of physical activity across the life-course can inform prevention strategies which may contribute to a reduction in pancreatic cancer. Future research is required to further explore the inverse associations in trajectories characterized by increased physical activity in younger adulthood and decreased physical activity in later life.

## Supplementary information

**Additional file 1: Table S1.** Odds ratio estimates for moderate physical activity levels throughout the life-course among Cases and Controls from Ontario, Canada. **Table S2.** Odds ratio estimates for vigorous physical activity levels throughout the life-course among Cases and Controls from Ontario, Canada

## Data Availability

Data are available from the Ontario Pancreas Cancer Study and Ontario Cancer Risk Factor Study; however, access restrictions apply (data transfer agreement required by Cancer Care Ontario, and REB approval would be required). Authors Steven Gallinger and Michelle Cotterchio may be contacted for any requests at steven.gallinger@uhn.ca and michelle.cotterchio@cancercare.on.ca

## Abbreviations

BMI: Body mass index
CI: Confidence Interval
MET: Metabolic equivalent of time
OCRF: Ontario Cancer Risk Factor Study
OPCS: Ontario Pancreas Study
OR: Odds ratio
