# Supplementary material for: Trajectories of physical activity, from young adulthood to older adulthood, and pancreatic cancer risk; a population-based case-control study in Ontario, Canada
Source: BMC Cancer. 2020 Feb 21;20:139. doi: 10.1186/s12885-020-6627-8 (PMC7035748; doi:10.1186/s12885-020-6627-8)
Supplement: Supplementary file 1 — Additional file 1: Table S1. Odds ratio estimates for moderate physical activity levels throughout the life-course among Cases and Controls from Ontario, Canada. Table S2. Odds ratio estimates for vigorous physical activity levels throughout the life-course among Cases and Controls from Ontario, Canada [file 12885_2020_6627_MOESM1_ESM.docx]

SUPPLEMENTAL TABLES

**Table S1:** Odds ratio estimates for moderate physical activity levels throughout the life-course among Cases and Controls from Ontario, Canada

|  | **< 65 years of age** | | | **≥ 65 years of age** | | |
| --- | --- | --- | --- | --- | --- | --- |
| Physical activity levels for various periods | Cases  N=145  (%) | Controls  N=724  (%) | OR^1^  (95% CI) | Cases  N=167  (%) | Controls  N=530  (%) | OR^1^  (95% CI) |
| Moderate activity level at age 20s and 30s  Rarely/Never  A few times per month (1/week)  2-4 times per week  >4 times per week | 5  19  32  44 | 3  20  35  42 | 1.00  0.65 (0.23, 1.83)  0.62 (0.23, 1.70)  0.70 (0.26, 1.90) | 12  19  32  37 | 6  20  30  43 | 1.00  0.53 (0.25, 1.10)  0.69 (0.34, 1.37)  0.50 (0.25, 0.98) |
| Moderate activity level at ages 40s and 50s  Rarely/Never  A few times per month (1/week)  2-4 times per week  >4 times per week  Age not reached | 8  24  33  31  4 | 7  22  39  31  2 | 1.00  1.00 (0.44, 2.31)  0.79 (0.35, 1.78)  0.90 (0.40, 2.04) | 8  20  37  34  0 | 8  24  35  33  0 | 1.00  0.77 (0.35, 1.73)  1.25 (0.58, 2.66)  1.12 (0.52, 2.42) |
| Moderate activity level 2 years ago  Rarely/Never  A few times per month (1/week)  2-4 times per week  >4 times per week | 10  13  34  43 | 8  17  37  38 | 1.00  0.50 (0.22, 1.15)  0.66 (0.32, 1.35)  0.81 (0.40, 1.65) | 10  11  38  41 | 8  15  37  40 | 1.00  0.64 (0.28, 1.47)  0.78 (0.38, 1.64)  0.95 (0.46, 1.97) |

1. Sex, alcohol consumption, smoking, vegetable consumption, fruit consumption, red meat consumption, family history of pancreatic cancer, race, education adjusted OR

**Table S2.** Odds ratio estimates for vigorous physical activity levels throughout the life-course among Cases and Controls from Ontario, Canada

|  | **< 65 years of age** | | | **≥ 65 years of age** | | |
| --- | --- | --- | --- | --- | --- | --- |
| Physical activity levels for various periods | Cases  N=145  (%) | Controls  N=724  (%) | OR^1^  (95% CI) | Cases  N=167  (%) | Controls  N=530  (%) | OR^1^  (95% CI) |
| Moderate activity level at age 20s and 30s  Rarely/Never or a few times per month  2-4 times per week  >4 times per week | 24  32  44 | 23  35  42 | 1.00  0.90 (0.53, 1.51)  1.01 (0.62, 1.67) | 31  32  37 | 27  30  43 | 1.00  1.07 (0.66, 1.74)  0.78 (0.49, 1.24) |
| Moderate activity level at ages 40s and 50s  Rarely/Never or a few times per month  2-4 times per week  >4 times per week  Age not reached | 32  33  31  4 | 28  39  31  2 | 1.00  0.79 (0.48, 1.28)  0.90 (0.55, 1.47) | 28  37  34  0 | 31  35  33  0 | 1.00  1.25 (0.58, 2.66)  1.12 (0.52, 2.42) |
| Moderate activity level 2 years ago  Rarely/Never or a few times per month  2-4 times per week  >4 times per week | 23  34  43 | 24  37  38 | 1.00  1.03 (0.61, 1.73)  1.26 (0.76, 2.10) | 21  38  41 | 23  37  40 | 1.00  1.51 (0.94, 2.44)  1.36 (0.82, 2.24) |

1. Sex, alcohol consumption, smoking, vegetable consumption, fruit consumption, red meat consumption, family history of pancreatic cancer, race, education adjusted OR
